# Mepolizumab and benralizumab in patients with severe asthma and a history of eosinophilic granulomatosis with polyangiitis

**DOI:** 10.3389/fmed.2024.1341310

**Published:** 2024-03-22

**Authors:** Charlene Desaintjean, Kaïs Ahmad, Julie Traclet, Mathieu Gerfaud-Valentin, Cecile-Audrey Durel, Jean-Charles Glerant, Arnaud Hot, François Lestelle, Sabine Mainbourg, Mouhamad Nasser, Pascal Seve, Ségolène Turquier, Gilles Devouassoux, Vincent Cottin

**Affiliations:** ^1^Department of Respiratory Medicine, National Reference Centre for Rare Pulmonary Diseases, Member of ERN-LUNG, Louis Pradel Hospital, Hospices Civils de Lyon, Lyon, France; ^2^Department of Internal Medicine, Hôpital Saint-Joseph Saint-Luc, Lyon, France; ^3^Pulmonary Function Tests Department, Louis Pradel Hospital, Hospices Civils de Lyon, Lyon, France; ^4^Department of Internal Medicine, Edouard Herriot Hospital, Hospices Civils de Lyon, Lyon, France; ^5^Department of Internal Medicine and Vascular Medicine, Lyon Sud Hospital, and Lyon Immunopathology Federation (LIFe), Hospices Civils de Lyon, Lyon, France; ^6^UMR 5558, Laboratoire de Biométrie et Biologie Evolutive, Claude Bernard University Lyon 1, Lyon, France; ^7^Department of Internal Medicine, Hôpital de la Croix Rousse, Hospices Civils de Lyon, Lyon, France; ^8^Research on Healthcare Performance (RESHAPE), INSERM U1290, Claude Bernard University Lyon 1, Lyon, France; ^9^Department of Respiratory Medicine, CIERA, Hôpital de la Croix Rousse, Hospices Civils de Lyon, Lyon, France; ^10^CRISALIS INSERM, F-CRIN Network, Toulouse, France; ^11^VirPath, INSERM U1111-CNRS UMR 5308-ENS de Lyon, Université Claude Bernard Lyon 1, Lyon, France; ^12^UMR 754, INRAE, Claude Bernard University Lyon 1, Lyon, France

**Keywords:** Interleukin-5, vasculitis, eosinophil, asthma, corticosteroids, granulomatosis

## Abstract

**Introduction:**

Asthma associated with eosinophilic granulomatosis with polyangiitis (EGPA) is often severe and corticosteroid-dependent, leading to significant morbidity. Mepolizumab and benralizumab are humanized monoclonal antibodies targeting interleukin 5 (IL-5) and its receptor, respectively. They have been shown to be effective in steroid-sparing in patients with severe eosinophilic asthma.

**Objective:**

Our aim was to evaluate the efficacy and safety of mepolizumab and benralizumab prescribed for severe asthma in patients with EGPA under “real-world” conditions.

**Methods:**

This was a retrospective analysis of patients with EGPA and persistent asthma who received either mepolizumab 100 or 300 mg administered every 4 weeks, or benralizumab 30 mg administered every 4 weeks for the initial 3 injections and followed by an injection every 8 weeks thereafter, whilst combined with oral glucocorticoids. The follow-up every 6 ± 3 months included an assessment of clinical manifestations, pulmonary function tests and eosinophil cell count. The primary outcome was the proportion of patients at 12 months receiving a daily oral dose of prednisone or equivalent of 4 mg or less with a BVAS of 0.

**Results:**

Twenty-six patients were included. After 12 months of treatment with mepolizumab or benralizumab, 32% of patients met the primary outcome and were receiving less than 4 mg of prednisone per day with a BVAS of 0. The median dose of prednisone was 10 mg per day at baseline, 9 mg at 6 months, and 5 mg at 12 months (*p* ≤ 0.01). At 12 months, 23% of patients were weaned off corticosteroids, while an increase or no change in dose was observed in 27% of patients. The median eosinophil count was significantly reduced from 365 cells/mm^3^ to 55 cells/mm^3^ at 6 months and 70 cells/mm^3^ at 12 months, respectively. No significant change was observed in FEV1. After 12 months of treatment, 14% of patients had had an average of 1 exacerbation of asthma, compared with 52% of patients before baseline. The tolerability profile was favorable.

**Conclusion:**

In this real-world study in patients with severe asthma and a history of EGPA asthma, mepolizumab and benralizumab had a significant steroid-sparing effect and reduced asthma exacerbation, but no significant effect on lung function.

## Introduction

Eosinophilic granulomatosis with polyangiitis (EGPA) belongs to the spectrum of anti-neutrophil cytoplasmic antibody (ANCA)-associated vasculitides predominantly affecting small to medium vessels (1). It is characterised by an association with asthma, ear, nose and throat (ENT) involvement, blood and tissue eosinophilia and systemic vasculitis manifestations (2).

Asthma is almost always present in EGPA. It is often severe and usually precedes systemic manifestations by several years (3). The treatment of EGPA is based on systemic glucocorticoids, and immunosuppressive agents in severe cases (4, 5). However, a long-term treatment with low-dose glucocorticoids is often necessary to prevent asthma flares and relapses of vasculitis and leads to significant morbidity (3, 6–9). Reducing treatment-related morbidity is a priority of novel treatment approaches (10).

Mepolizumab, a humanized anti-interleukin-5 (IL-5) monoclonal antibody, reduces blood eosinophil counts and has demonstrated benefit at a dose of 100 mg subcutaneously every 4 weeks in patients with severe eosinophilic asthma (11, 12). In the phase 3, randomised controlled trial MIRRA, which enrolled patients with relapsing or refractory EGPA, a regimen of mepolizumab 300 mg every 4 weeks increased the duration of remission and resulted in a higher proportion of participants in remission than placebo did (13). Mepolizumab treatment was also associated with a greater proportion of patients with a daily dose of glucocorticoids of less than 4 mg per day during weeks 48 through 52, suggesting a steroid-sparing effect (13), consistent with observational data (14). Mepolizumab use was approved by the Food and Drug Administration (FDA) and by the European Medicine Agency (EMA) to treat relapsing or refractory EGPA.

Benralizumab is a monoclonal antibody targeting the IL-5 receptor-alpha. Using a regimen of 30 mg every 8 weeks after the first 3 injections every 4 weeks, benralizumab reduces blood eosinophil counts, and significantly reduces annual asthma exacerbation rates in patients with severe, uncontrolled asthma with blood eosinophils 300 cells/mm^3^ or greater (15, 16). A steroid-sparing effect of benralizumab was reported in small-size, retrospective cohorts (17) and prospective studies of patients with EGPA and severe asthma (18, 19). Comparable results were reported in a pilot study using reslizumab, another monoclonal antibody bindings to the alpha chain of the IL-5 receptor (20). However, few studies were conducted in patients with severe asthma and a history of EGPA.

In this retrospective study, we assessed the benefit of mepolizumab and benralizumab on the use of oral glucocorticoids, respiratory manifestations and lung function, and their tolerability by patients with asthma following EGPA, in real-life settings.

## Patients and methods

### Study design and patients

We conducted a retrospective monocentric study in a large tertiary care institution (Hospices Civils in Lyon). Cases enrolled in the study were admitted to one of the six departments of pulmonology or internal medicine of the institution between 1995 and July 2021, who were prescribed mepolizumab and/or benralizumab for severe persistent asthma, had a history of EGPA, and had been followed-up at least 12 months after the initiation of treatment. Patients who were prescribed mepolizumab or benralizumab were identified using the electronic patient files and the database of the hospital pharmacy (reslizumab was not available), and patient charts were reviewed manually. Patients had a clinical diagnosis of EGPA which satisfied either the inclusion criteria used in the MIRRA study (13) or the 1990 American College of Rheumatology (21) or the 2022 American College of Rheumatology / European Alliance of Associations for Rheumatology classification criteria (22), namely: (1) asthma; (2) peripheral blood eosinophilia >1,000/mm^3^ or 10% and (3) the presence of two or more criteria that are typical of EGPA. Patients who had granulomatosis with polyangiitis, microscopic polyangiitis, hypereosinophilic syndrome, or idiopathic chronic or acute eosinophilic pneumonia, eosinophilia related to parasite infections or malignant tumors, were excluded, as well as pregnant or breastfeeding women.

### Procedures and data collection

Data was collected at the diagnosis of EGPA, at the initiation of mepolizumab or benralizumab, and at 6-month and 12-month follow-up visits. The treatment was left to the discretion of the attending physician including for tapering oral glucocorticoids. Data was collected during each visit by the caring physician using a standardised case-report form. Assessments included demographics, clinical manifestations, and biological data including blood and alveolar eosinophil counts, presence of ANCA. Lung function tests were performed following international guidelines and with standardised protocols. We assessed forced expiratory volume in 1 s (FEV1), forced vital capacity (FVC) and the FEV1/FVC ratio before bronchodilators and then 10 min after the inhalation of a short-acting beta-2 agonist. All values were expressed as percentage of predicted values, except for the FEV1/FVC ratio which was expressed as an absolute percentage and mean standard deviation. Reversibility was defined by an improvement of FEV1 with short-acting beta-2 agonist of 200 millilitres or more with 12% improvement or greater compared to the pre-bronchodilator FEV1.

Vasculitis activity was assessed using the Birmingham Vasculitis Activity Score (BVAS) from 2003 (0 → 63). Medications taken by the patient were extracted from electronic patients’ files and prescriptions, including daily prednisone dose and concomitant therapy. Severe asthma exacerbations were assessed per 6 month-periods, as well as any significant event including respiratory infections. Severe asthma exacerbations were defined as the worsening of respiratory symptoms (dyspnea, wheezing, cough) necessitating the initiation or increase of systemic glucocorticoids, visit to the emergency department, or non-elective hospitalisation. In order to monitor safety, adverse effects were assessed at every visit.

### Outcome

The primary efficacy outcome was the proportion of patients who had a daily prednisone dose reduced to 4 mg or less at 12 months, and a BVAS score of 0, as per the MIRRA trial (13). Secondary efficacy outcomes were the proportion of patients who had a daily prednisone dose reduced to 4 mg or less at 6 months; the proportion of patients with a decrease of the median corticosteroid dose of 50% or more at 6 months and at 12 months; a significant decrease in the oral corticosteroid dose at 6 and 12 months; the percentage of patients weaned of oral glucocorticoids; a decrease in eosinophil blood count; and the improvement of lung function (FEV1 before bronchodilators, FEV1/FVC). We also evaluated the tolerability and safety of biologics over 12 months.

### Statistical analysis

Descriptive results are expressed as median (interquartile) for quantitative variables and as number (percentage) for qualitative variables. Baseline measures were compared to those at 6 and 12 months after the initiation of mepolizumab or benralizumab, using chi-squared or the Fischer’s exact test for the qualitative variables. The Wilcoxon signed-rank test was applied to compare pre- and post-treatment lung function, and the Mann–Whitney *U* test was used to compare measures between groups. All tests were two sided, and statistical significance was set at *p* < 0.05 (two-tailed). IBM SPSS Statistics for Windows, Version 25.0 (IBM Corp., Armonk, NY) was used for statistical analyses. Graphs were created using GraphPad Prism.

### Ethics

This study was conducted with respect to the Declaration of Helsinki. It was approved by the ethics committee of the Hospices Civils de Lyon and was registered with the national data protection agency (Commission Nationale de l’Informatique et des Libertés, number 21-5484). According to the legislation in place at the time of the study, informed consent signature was waived, but each patient was informed by a written letter and could object to the use of their personal data.

## Results

### Study population at EGPA diagnosis

A total of 26 patients were included (14 women and 12 men) with a median age of 49.5 years at EGPA diagnosis.

Clinical manifestations at the time of EGPA diagnosis are showed in Table 1. The median blood eosinophil count was 41% or 5,970 cells/mm^3^. Apart from asthma, the most frequent manifestations were sinonasal abnormalities (including chronic rhinitis, sinusitis, polyposis, and nasal obstruction) (76.9% of patients), myocarditis defined by elevated troponine levels with suggestive echocardiography or cardiac magnetic resonance imaging (38.5%), mononeuritis multiplex (30.8%), pericardial effusion at echocardiography (30.8%), fever (30.8%), weight loss (26.9%), and skin involvement (23.1%). Eosinophilic pneumonia with non-fixed pulmonary infiltrates and ground-glass attenuation was present in 84.6% of patients on computed-tomography (CT) scan. The median alveolar eosinophil count was 52%.

**Table 1:** Main characteristics at EGPA diagnosis.

	All patients (*n* = 26)
Median age—years (range)	49.5 (21–77)
Female sex—no (%)	14 (53.8)
Median body mass index—kg/m^2^ (range)	24.9 (18–34.9)
Current smoker—no (%)	1 (4.2)
Former smoker—no (%)	3 (12.5)
BVAS – median (IQR)	14 (11.3–18)
*Clinical and radiological manifestations at EGPA diagnosis*	
Asthma—no (%)	26 (100)
Sinonasal abnormality—no (%)	20 (76.9)
Eosinophilic pneumonia—no (%)	22 (84.6)
Alveolar hemorrage—no (%)	0 (0)
Mononeuritis multiplex—no (%)	8 (30.8)
Polyneuritis—no (%)	0 (0)
Central nervous system involvement—no (%)	0 (0)
Myocardial involvement on MRI or echocardiography—no (%)	10 (38.5)
Pericarditis—no (%)	8 (30.8)
Coronary arteritis—no (%)	0 (0)
Glomerulonephritis—no (%)	2 (7.7)
Interstitial nephritis—no (%)	1 (3.8)
Skin involvement—no (%)	6 (23.1)
*Biology*	
Median blood eosinophil count—% (IQR)	41 (23–54)
Median blood eosinophil count—cells/mm^3^ (IQR)	6.0 (3.3–10.0)
Median alveolar eosinophil count—% (IQR)	52 (43–56)
missing data—no (%)	17 (65.4)
ANCA status positive—no (%)	5 (25)
Specificity MPO—no (%)	5 (25)
specificity PR3—no (%)	0 (0)
Biopsy evidence of vasculitis—no (%)	9 (34.6)
*Lung function tests*	
Median FEV1/FVC ratio (range)	70 (36–87)
Median FEV1—percent of predicted value before bronchodilation (range)	91 (22–117)
Median FEV1 before bronchodilation, liters (range)	2.6 (1.0–4.0)
Significative reversibility—no (%)	1 (6.7)
missing data—no (%)	11 (42.3)
*Treatments*	
Immunosuppressive treatment between diagnosis and baseline—no (%)	18 (69.2)
Omalizumab between diagnosis and baseline—no (%)	4 (15.4)

All patients had persistent asthma despite daily oral glucocorticoids, inhaled corticosteroids, and long-acting bronchodilators. Thirteen patients had an obstructive ventilatory defect (FEV1/FVC ratio <0.70). The median FEV1/FVC ratio was 0.70. The median FEV1 was 2.59 L (91% of predicted value) before bronchodilation. The FEV1 was reversible in only one patient. Three patients were former smokers, one was a current smoker, and the others had never smoked.

Perinuclear ANCAs were found in 5 patients (19.2%), all with myeloperoxidase specificity. The vasculitis had been confirmed by biopsy in 34.6% of patients: skin (*n* = 3), pulmonary (*n* = 2), nasal cavity, muscle, gall bladder, renal (*n* = 1 each). Eighteen patients (69.2%) had received an immunosuppressive therapy (Supplementary Table S1).

### Characteristics at initiation of IL5/IL5R therapy

At the time of initiation of anti-IL5/IL5R therapy (thereafter referred to as baseline), the median age was 58 years (range 22–78) 12 months. The median BVAS was 1 (range 0–2). All the patients had a symptomatology of severe asthma, and 52% had at least one severe asthma exacerbation per 6 months. Twelve patients (46%) had sinonasal abnormalities; 4 (17%) had migrating pulmonary infiltrates. None of the patients had neurological, cardiac, renal, skin, or gastrointestinal involvements at baseline. The median blood eosinophil count was 4.5% or 365 cells/mm^3^.

At baseline, 17 patients (68%) had airflow obstruction. The median FEV1/FVC ratio was 0.65, and the median FEV1 before bronchodilation was 85% of predicted. There was a significant reversibility of FEV1 in 5 patients (33%). All patients were receiving 4 mg or more of oral prednisone, with a median daily dosage of 10 mg (interquartile range 7.6–20 mg). All patients were also receiving long-acting bronchodilators and inhaled corticosteroids. Seven patients (27%) were still receiving immunosuppressive treatment (5 receiving azathioprine, and 2 rituximab).

### Treatment

Mepolizumab/benralizumab treatment was introduced after a median duration of 5.5 years after the EGPA diagnosis. Fifteen patients were treated with subcutaneous injections of mepolizumab 100 mg every 4 weeks, 1 patient received mepolizumab 300 mg every 4 weeks, and 10 patients were treated with subcutaneous injections of benralizumab 30 mg administered at baseline, week 4, week 8, then every 8 weeks. Treatment with benralizumab was discontinued after 9 months in one patient who became pregnant.

### Outcome

**Figure 1:**
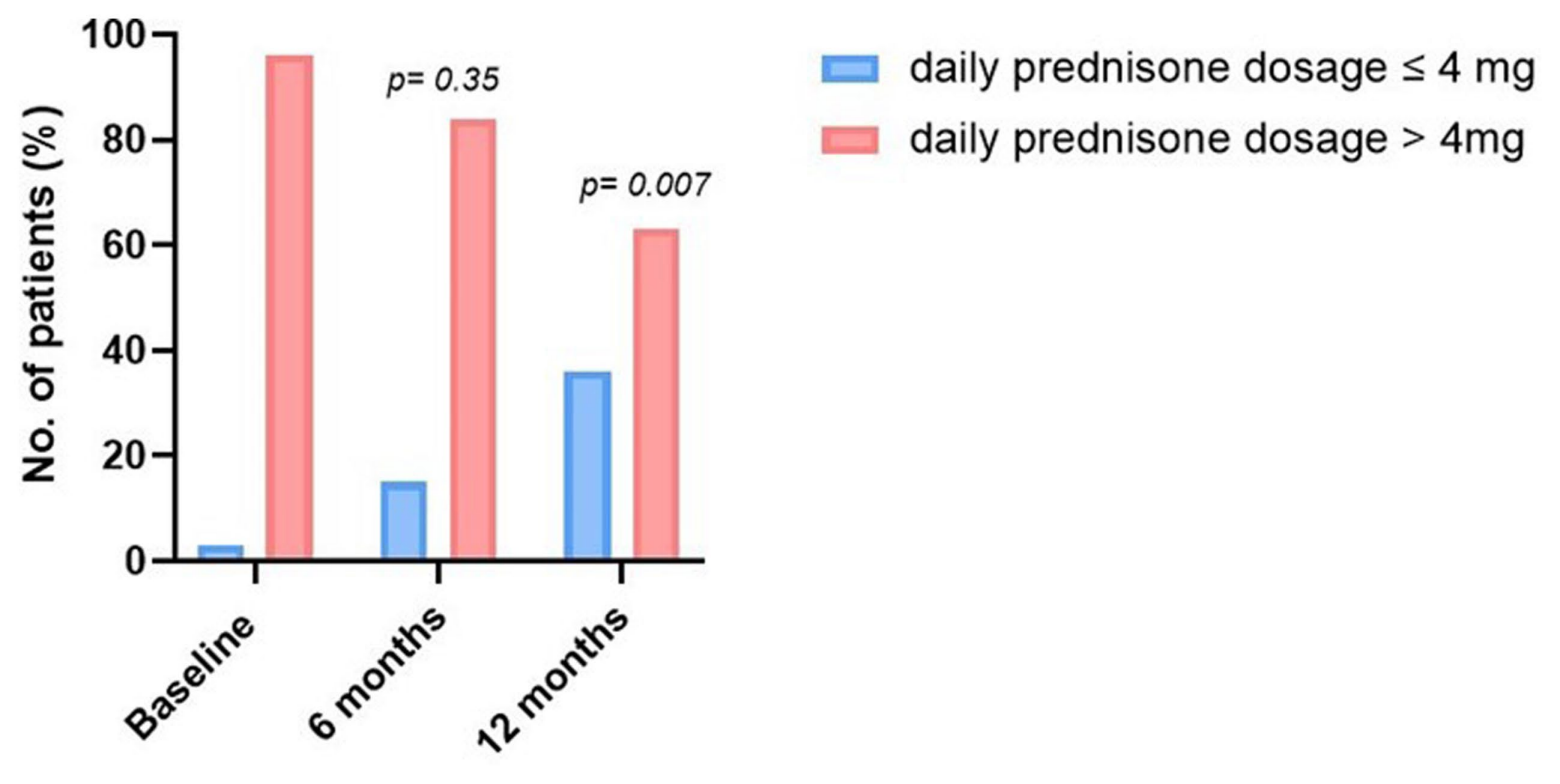
Evolution of the proportion of patients according to the daily dosage of oral glucocorticoids during the follow-up.

The median duration of follow-up was 12 months for efficacy and 721 days for safety. At 12 ± 3 months, 32% of patients (7/22) had reached the primary endpoint and had reduced the daily prednisone dosage to 4 mg or less (Figure 1) with a BVAS score of 0. For the second efficacy outcomes, 15% of patients (4/26) had reduced the daily prednisone dosage to 4 mg or less at 6 ± 3 months (Supplementary Figure S1). The median prednisone daily dosage significantly decreased from 10 mg at baseline to 9 mg at 6 ± 3 months (*p* = 0.0032) and further to 5 mg at 12 ± 3 months (*p* = 0.0052) (Table 2; Supplementary Table S2). At 12 months, 5 patients (23%) had discontinued oral glucocorticoid therapy. The daily dose of oral glucocorticoids was reduced by 50% or more in 31% of the patients at 6 months (*n* = 8) and in 55% at 12 months (*n* = 12), but was unchanged or increased in 6 patients (27%) at 12 months (Supplementary Table S3).

**Table 2:** Main outcome variables (*n* = 26).

	Baseline	6 ± 3 months	12 ± 3 months
Median body mass index—kg/m^2^ (range)	24.7 (18–33.9)	24.9 (22.4–29.1)	25.3 (22.1–28.5)
*Clinical manifestations*			
Dyspnoea—no (%)	15 (60)	8 (31)	4 (19)^*^
Sinonasal abnormality—no (%)	12 (46)	10 (38)	9 (41)
Median BVAS (IQR)	1 (0–2)	0.5 (0–1)^*^	1 (0–1)
*Pulmonary exacerbations*			
≥1 asthma severe exacerbation/6 months—no (%)	13 (52)	4 (16)^*^	3 (14)^*^
Infections—no (%)	5 (24)	4 (16)	5 (24)
*Biological markers*			
Median blood eosinophil count—% (IQR)	4.5 (1.9–7.1)	0.5 (0–1.9)^*^	0.6 (0–1.7)^***^
Median blood eosinophil count—cells/mm^3^ (IQR)	365 (158–570)	55 (0–155)^*^	70 (5–145)^***^
Median CRP—mg/liter (IQR)	2 (1.9–2.9)	1.9 (1–3.1)	2 (1.4–7)
*Pulmonary function tests*			
Median FEV1/ FVC—absolute % (IQR)	65 (56–72)	69 (61–73)^**^	67 (58–77)
Median FEV1 before BD—% predicted (IQR)	85 (67–101)	90 (74–103)	72 (65–91)
Median FEV1 before BD—liter (IQR)	2.4 (2.0–2.9)	2.6 (1.7–2.8)^*^	2 (1.8–2.8)
Significant reversibility—no (%)	5 (33)	1 (11)	3 (30)
*Treatments*			
Median prednisone dosage—mg per day (IQR)	10 (7.6–20)	9 (5–10)^**^	5 (2–10)^**^
Immunosuppressive agents—no (%)	7 (27)	6 (23)	6 (23)

^*^p ≤ 0.05 vs. baseline, ^**^p ≤ 0.01 vs. baseline, and ^***^p ≤ 0.001 vs. baseline.

**Figure 2:**
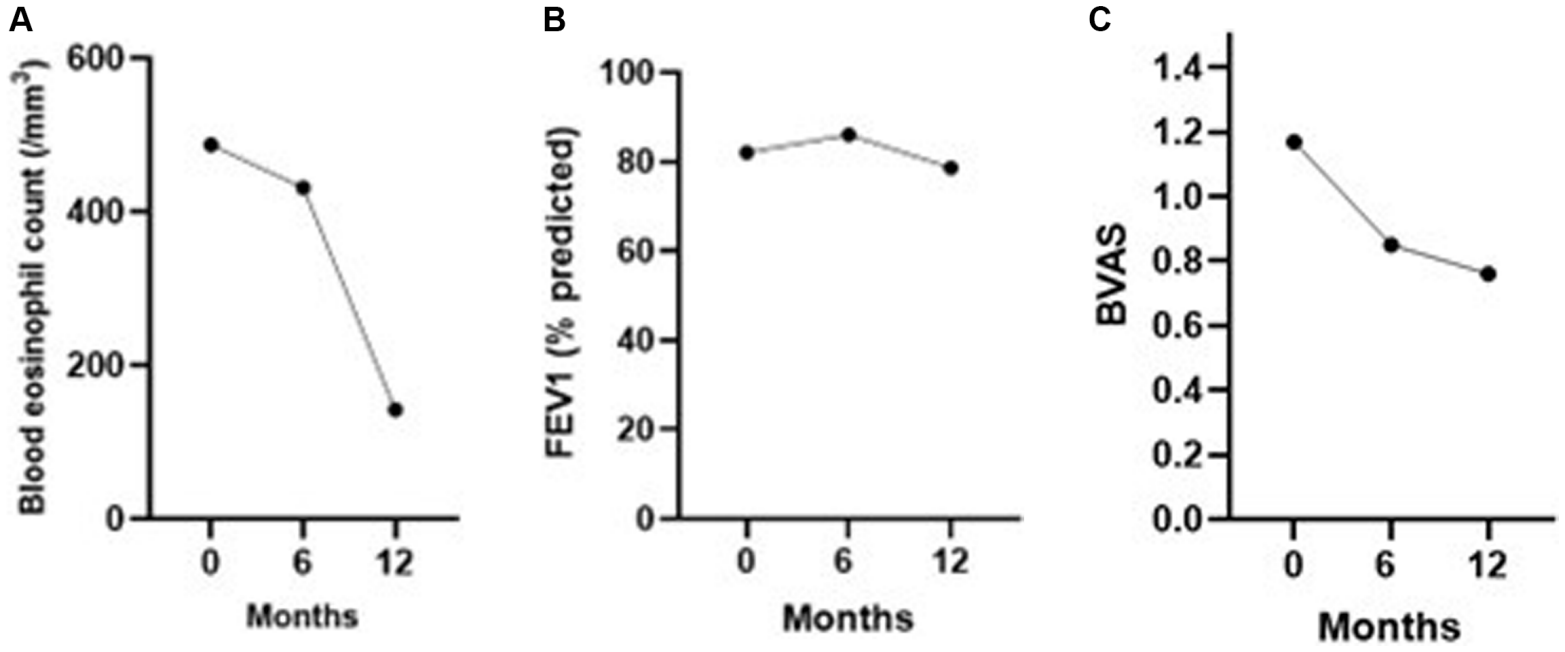
**(A)** Mean blood eosinophil count, **(B)** mean FEV1 (% predicted), and **(C)** mean BVAS during the 12 months of follow-up after anti-IL5/IL5(R) initiation.

The median blood eosinophil count significantly decreased during the follow-up, from 365 cells/mm^3^ at baseline to 55 cells/mm^3^ at 6 ± 3 months, and 70 cells/mm^3^ at 12 months (*p* = 0.00033) (Figure 2A). Asthma exacerbations were less frequent (14% versus 52%). No significant changes were observed in the rate of exertional dyspnea, sinonasal manifestations, FEV1, or use of immunosuppressive agents.

Seven patients (26.9%) discontinued prematurely anti-IL-5/Rα therapy, after a mean of 16.6 months: mepolizumab 100 mg (*n* = 4), mepolizumab 300 mg (*n* = 1), benralizumab 30 mg (*n* = 2). The reasons were: a perceived lack of efficacy (*n* = 5), a vasculitis flare (*n* = 1 with polyarthritis), and acute eosinophilic pneumonia (*n* = 1). The lack of efficacy was defined as a worsening of asthma (*n* = 3) or sinusitis (*n* = 1), or persistent blood eosinophilia with no change in the need of oral glucocorticoids. Four patients with response considered insufficient to mepolizumab switched to benralizumab, and one switched from benralizumab to mepolizumab.

Comparison of the efficacy of anti-IL-5/Rα biologics was limited by the small sample size. At 12 months, 4/13 patients (30.8%) receiving mepolizumab and 4/9 patients (44.4%) on benralizumab had 4 mg or less of oral glucocorticoids per day, respectively (Supplementary Figure S2). The median prednisone dosage at 12 months was 5 mg for each group. The median blood eosinophil count was 80 cells/mm^3^ in mepolizumab group and 0 cells/mm^3^ in benralizumab group at 12 months.

### Safety

The tolerability of anti-IL5/Rα biologics was unremarkable. No serious adverse event and no anaphylaxis were reported (Supplementary Table S4). The most frequent adverse effects were headache (15.4%) with mepolizumab (*n* = 3) or benralizumab (*n* = 1). While he was treated by mepolizumab and had been weaned off oral glucocorticoids, a patient developed an acute eosinophilic pneumonia, and was switched to benralizumab.

## Discussion

In this real-life study of patients with severe eosinophilic asthma and a history of EGPA, we found that treatment with mepolizumab or benralizumab was associated with a reduction in the daily dose of oral glucocorticoids, asthma flares, and in blood eosinophil cell counts, without safety alerts. No significant change in lung function was observed at 12 months.

Optimising the management of EGPA is a real therapeutic challenge since long-term glucocorticoids lead to significant morbidity, even when using “low” doses (23), including osteoporosis, cardiovascular and metabolic manifestations (24, 25). At 12 months, 36% of the patients were receiving 4 mg of prednisone per day or less; 55% had reduced their daily dose of glucocorticoids by 50% or more, and 23% had discontinued oral glucocorticoids. The median prednisone daily dose decreased from 10 mg at baseline to 9 mg at 6 months and 5 mg at 12 months. Our findings support the results of clinical trials demonstrating a glucocorticoid-sparing effect of mepolizumab and benralizumab in the setting of EGPA (13) and of severe eosinophilic asthma (11, 16). Comparable results were reported in a prospective open-label pilot study of benralizumab in 10 patients with eosinophilic asthma and a history of EGPA (18), with a reduction of the median glucocorticoid daily dose from 15 mg to 2 mg. Preliminary results from the MANDARA trial demonstrated the non-inferiority of benralizumab 30 mg every 4 weeks compared with mepolizumab 300 mg every 4 weeks, and suggested a greater glucocorticoid-sparing effect with benralizumab,^1^ as suggested by our cohort. A steroid-sparing effect of mepolizumab or benralizumab was also suggested in several multicenter retrospective studies (14, 26–28). Two patients had persisting eosinophilia despite biologics, raising the question of compliance to treatment.

However, it should be noted that 27% of our patients had an increase or no change in the dose of prednisone. In the MIRRA trial, 47% of participants receiving mepolizumab did not achieve complete remission (13). Predictors of response to anti-IL5/Rα biologics have not been identified in EGPA. In addition, no significant improvement in FEV1 was observed. Consistently, in the study by Nair et al. (16) in severe asthma, no significant effect of benralizumab on FEV1 was found. It is to be noted that only 5 patients (33%) had a significant reversibility at baseline, consistent with previous findings from our center in EGPA (6).

The doses of anti-IL-5/Rα biologics used in our study were those used in severe eosinophilic asthma (16, 23), and only one patient received mepolizumab 300 mg, the approved and recommended regimen to treat relapsing or active, non-severe EGPA (4), or the hypereosinophilic syndrome (29). In a European observational study of 191 patients with EGPA, mepolizumab 100 mg and 300 mg had a comparable efficacy (14), however the two regimens have not been compared prospectively. Our findings suggest that mepolizumab 100 mg every 4 weeks could be an acceptable regimen in patients with asthma in the setting of EGPA (3, 14, 30–35). Consistently, a recent guideline stated that an initial dosage of mepolizumab 100 mg every 4 weeks can be used for remission maintenance in EGPA; this dosage can subsequently be titrated up to 300 mg every 4 weeks in patients with an unsatisfactory response to treatment (36).

Only 25% of our patients had ANCAs at diagnosis of EGPA, compared to 30%–40% in the literature (37, 38). This is consistent with our patients being predominantly referred to departments of respiratory medicine with an ANCA-negative, “eosinophilic-driven” phenotype of EGPA, and the use of anti-IL-5/Rα biologics in severe asthma. Conceivably, treatment strategies might be adapted to the EGPA phenotype (39). The potential role of mepolizumab as a first-line treatment of EGPA, and the benefit of benralizumab versus mepolizumab, are currently being evaluated (NCT05030155, NCT04157348).

While anti-IL-5 /Rα biologics were generally well tolerated, headache was the most frequently reported adverse event, consistent with previous findings (11).

The strength of this real-world study is to include a relatively large number of consecutive patients from a single institution. The limitations of the study are inherent to the retrospective design. However, patients were followed-up regularly, with a single electronic chart system, facilitating data extraction and review, and limiting the amount of missing data. Quality of life questionnaire or clinical assessment scores were not available, and the decrease in the dosage of glucocorticoids was not standardised. However, most patients were followed in a respiratory department, which explains the large number of pulmonary function tests available and the particular emphasis placed on asthma control and steroid-sparing.

In conclusion, this real-life observational study supports the efficacy of mepolizumab and benralizumab as steroid-sparing agents in patients with EGPA and severe asthma, with a favorable tolerability profile.

## Data availability statement

The raw data supporting the conclusions of this article will be made available by the authors, without undue reservation.

## Ethics statement

The studies involving humans were approved by Ethics Committee of the Hospices Civils de Lyon, registered with the national data protection agency (Commission Nationale de l’Informatique et des Libertés, number 21-5484). The studies were conducted in accordance with the local legislation and institutional requirements. The ethics committee/institutional review board waived the requirement of written informed consent for participation from the participants or the participants’ legal guardians/next of kin because this was in line with the legislation in France on retrospective studies on existing data.

## Author contributions

CD: Data curation, Formal analysis, Investigation, Writing – original draft. KA: Validation, Writing – review & editing. JT: Validation, Writing – review & editing. MG-V: Validation, Writing – review & editing. C-AD: Validation, Writing – review & editing. J-CG: Validation, Writing – review & editing. AH: Validation, Writing – review & editing. FL: Validation, Writing – review & editing. SM: Validation, Writing – review & editing. MN: Validation, Writing – review & editing. PS: Validation, Writing – review & editing. ST: Validation, Writing – review & editing. GD: Validation, Writing – review & editing. VC: Conceptualization, Methodology, Supervision, Validation, Writing – review & editing.

## Footnotes

^1^ https://www.astrazeneca.com/media-centre/press-releases/2023/fasenra-phase-iii-egpa-trial-met-primary-endpoint.html
