# Supplementary material for: Mepolizumab and benralizumab in patients with severe asthma and a history of eosinophilic granulomatosis with polyangiitis
Source: Front Med (Lausanne). 2024 Mar 22;11:1341310. doi: 10.3389/fmed.2024.1341310 (PMC10998444; doi:10.3389/fmed.2024.1341310)

**Electronic supplement**

**e-Table 1**. Immunosuppressive treatment received prior to initiation of anti-IL5/IL5R therapy (baseline).

|  | **N (%)** |
| --- | --- |
| Prednisone or prednisolone | 26 (100) |
| Azathioprine | 13 (50) |
| Cyclophosphamide (IV pulses) | 9 (35) |
| Rituximab | 1 (4) |
| Methotrexate | 3 (12) |

**e-Table 2.** Main outcome variables (n=26), with p values.

| **Outcome variables  (n=26)** | **at baseline** | **6 ± 3 months** | **P-value** | **12 ± 3 months** | **P-value** |
| --- | --- | --- | --- | --- | --- |
| Median body mass index – kg/m² (range) | 24.7(18-33.9) | 24.9(22.4-29.1) | p=0.7938 | 25.3(22.1-28.5) | p=0.5872 |
| *Clinical manifestations* | | | | |  |
| Dyspnoea – no (%) | 15(60) | 8(31) | p=0.0877 | 4(19)* | p=0.0225 |
| Sinonasal abnormality – no (%) | 12(46) | 10(38) | P=0.7789 | 9(41) | p=1 |
| Median BVAS (IQR) | 1(0-2) | 0.5(0-1)* | p=0.0291 | 1(0-1) | p=0.0519 |
| *Pulmonary exacerbations* | | | | |  |
| ≥ 1 asthma severe exacerbation/6months – no (%) | 13(52) | 4(16)* | p=0.0156 | 3(14)* | p=0.0105 |
| Infections – no (%) | 5(24) | 4(16) | p=0.7252 | 5(24) | p=0.9246 |
| *Biological markers* | | | | |  |
| Median blood eosinophil count - % (IQR) | 4.5(1.9-7.1) | 0.5(0-1.9)* | p=0.0112 | 0.6(0-1.7)*** | p=0.0002 |
| Median blood eosinophil count - cells/mm3 (IQR) | 365(158-570) | 55(0-155)* | p=0.0179 | 70(5-145)*** | p=0.0003 |
| Median CRP – mg/liter (IQR) | 2(1.9-2.9) | 1.9(1-3.1) | p=0.8330 | 2(1.4-7) | p=0.3613 |
| *Functional respiratory tests* | | | | |  |
| Median FEV1/ FVC - absolute % (IQR) | 65 (56-72) | 69(61-73)** | p=0.0065 | 67(58-77) | p=0.2095 |
| Median FEV1 before BD - % predicted (IQR) | 85(67-101) | 90(74-103) | p=0.1835 | 72(65-91) | p=0.9793 |
| Median FEV1 before BD – liter (IQR) | 2.38(2-2.85) | 2.6(1.7-2.8)* | p=0.0495 | 2(1.8-2.8) | p=0.7761 |
| Significant reversibility – no (%) | 5(33) | 1(11) | p=0.5765 | 3(30) | P=1 |
| *Treatments* | | | | |  |
| Median prednisone dosage - mg per day (IQR) | 10 (7.6-20) | 9 (5-10)** | p=0.0032 | 5 (2-10)** | p=0.0052 |
| Immunosuppressive agents – no (%) | 7(27) | 6(23) | p=1 | 6(23) | P=1 |

**e-Table 3**. Reduction of daily prednisone dosage from baseline*

|  | at 6 months ±3 | | at 12 months ±3 | |
| --- | --- | --- | --- | --- |
|  | no. of patients (n=26) | % | no. of patients (n=22) | % |
| 100 % : discontinuation of glucocorticoids | 4 | 15 | 5 | 23 |
| ≥90 % | 4 | 15 | 6 | 27 |
| ≥75 % | 5 | 19 | 7 | 32 |
| ≥50 % | 8 | 31 | 12 | 55 |
| ≥25 % | 13 | 50 | 14 | 64 |
| > 0 % | 17 | 65 | 16 | 78 |
| an increase or no change in dose | 9 | 35 | 6 | 27 |

** missing data for 4 patients at 12 months*

**e-Table 4**. Adverse events

| Number of patients (%) | During first year of follow-up | >1 year of follow-up |
| --- | --- | --- |
| Cardiovascular adverse event | 0 (0) | 0 (0) |
| Serious adverse event | 0 (0) | 0 (0) |
| Fatal event | 0 (0) | 0 (0) |
| Anaphylaxis | 0 (0) | 0 (0) |
| Headache | 4 (15.4) | 0 (0) |
| Asthenia | 1 (3.8) | 0 (0) |
| Arthralgia | 0 (0) | 0 (0) |
| Worsening asthma | 2 (7.7) | 1 (4) |
| Worsening sinusitis | 1 (3.8) | 0 (0) |
| Systemic flare | 1 (3.8) | 0 (0) |
| Eosinophilic pneumonia | 0 (0) | 1 (4) |

**e- Figure 1**. Evolution of the daily dosage of oral glucocorticoids per patient at 6 and 12 months of

follow-up.


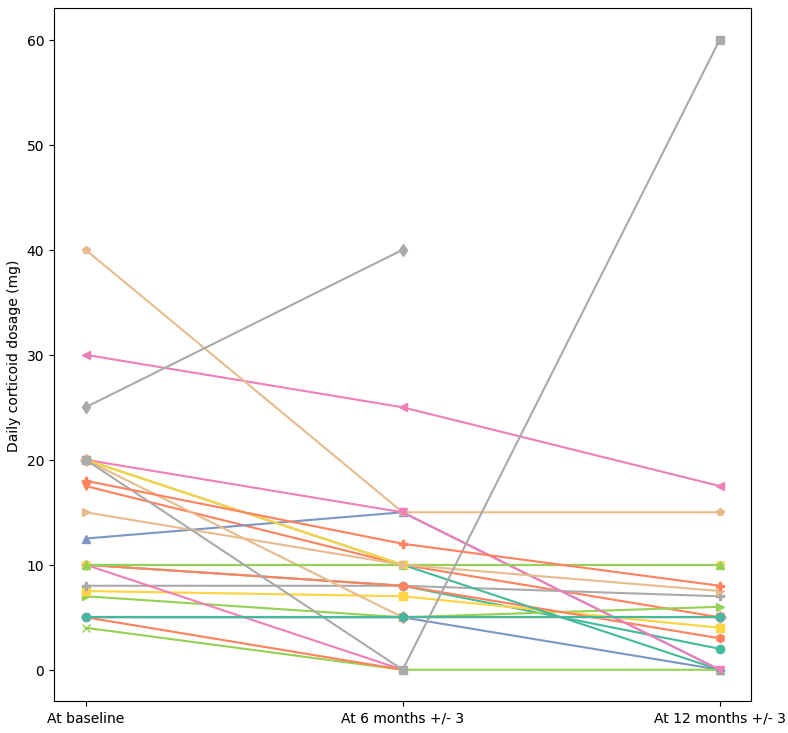


**e-Figure 2**. Daily dosage of glucocorticoids per patient during the follow-up: in the group treated by mepolizumab and in the group treated by benralizumab.


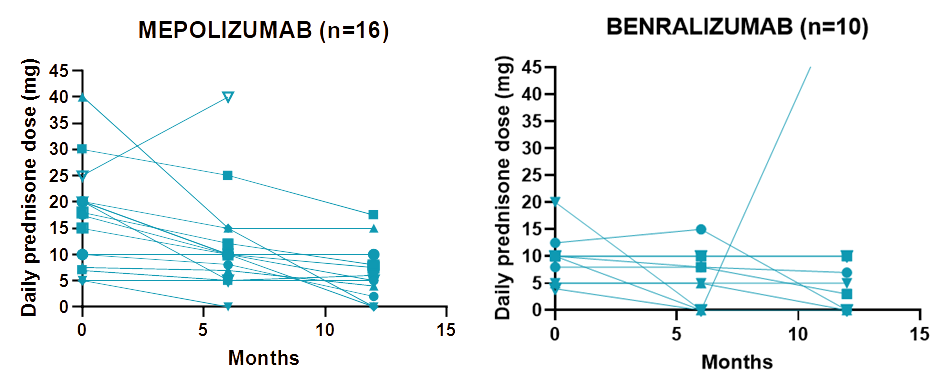

Supplement: Supplementary file 1 [file Data_Sheet_1.docx]
